# Supplementary material for: Circadian neurons in the paraventricular nucleus entrain and sustain daily rhythms in glucocorticoids
Source: Nat Commun. 2021 Oct 1;12:5763. doi: 10.1038/s41467-021-25959-9 (PMC8486846; doi:10.1038/s41467-021-25959-9)
Supplement: Supplementary file 1 — Supplementary Information [file 41467_2021_25959_MOESM1_ESM.pdf]

Supplementary Figures

Circadian neurons in the paraventricular nucleus entrain and sustain daily rhythms in glucocorticoids

Jeff R. Jones, Sneha Chaturvedi, Daniel Granados-Fuentes, and Erik D. Herzog

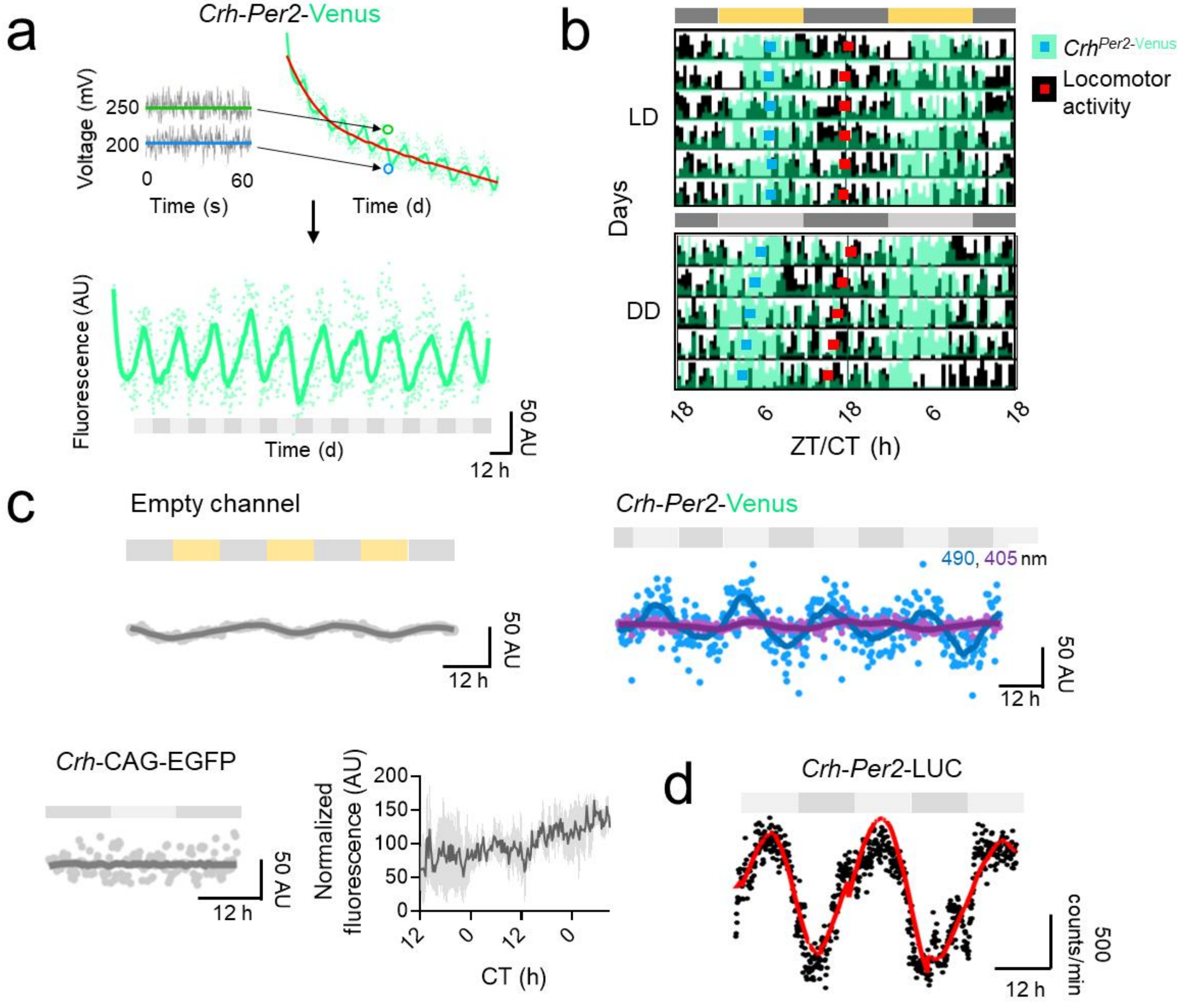

Supplementary Figure 1, related to Figure 1. Controls illustrating the specificity of *Per2*-Venus recording in PVN^{CRH} neurons. a) Schematic for fiber photometry analysis of *Per2*-Venus fluorescence recorded over 10 days. For details, see methods. **b**) A representative LD and DD trace from an animal in which we recorded both *Per2*-Venus (light green) and locomotor activity (via infrared, black). Acrophases are depicted by blue (*Per2*-Venus) and red (locomotor activity) squares. **c**) Representative control traces recorded for 60 s every 15 min. *Top*, fluorescence values (gray dots) recorded from a fiber without a mouse over 3 d in LD. *Bottom*, fluorescence values recorded *in vivo* in DD for 36 h from the PVN of a representative (*left*), and 5 (*right*), *Crh*-CAG-EGFP mice (mean ±SEM). *Right*, Five-day fluorescence traces recorded *in vivo* from the PVN of an individual *Crh-Per2*-Venus mouse excited with 490 nm and 405 nm light (blue and purple dots, respectively). The relative excitation of mVenus fluorescence at 490 nm is 0.37 and at 405 nm is 0.01 (fpbase.org). **d**) *In vivo* photon counts (black dots) recorded with a photomultiplier tube every 5 min in DD from an individual *Crh*-Cre; *Per2*-DIO-luc mouse that expressed *Per2*-driven luciferase in PVN^{CRH} neurons. Data were smoothed with a 4 h Savitzky-Golay filter (red line). Light gray and dark gray bars represent subjective day and night, respectively.

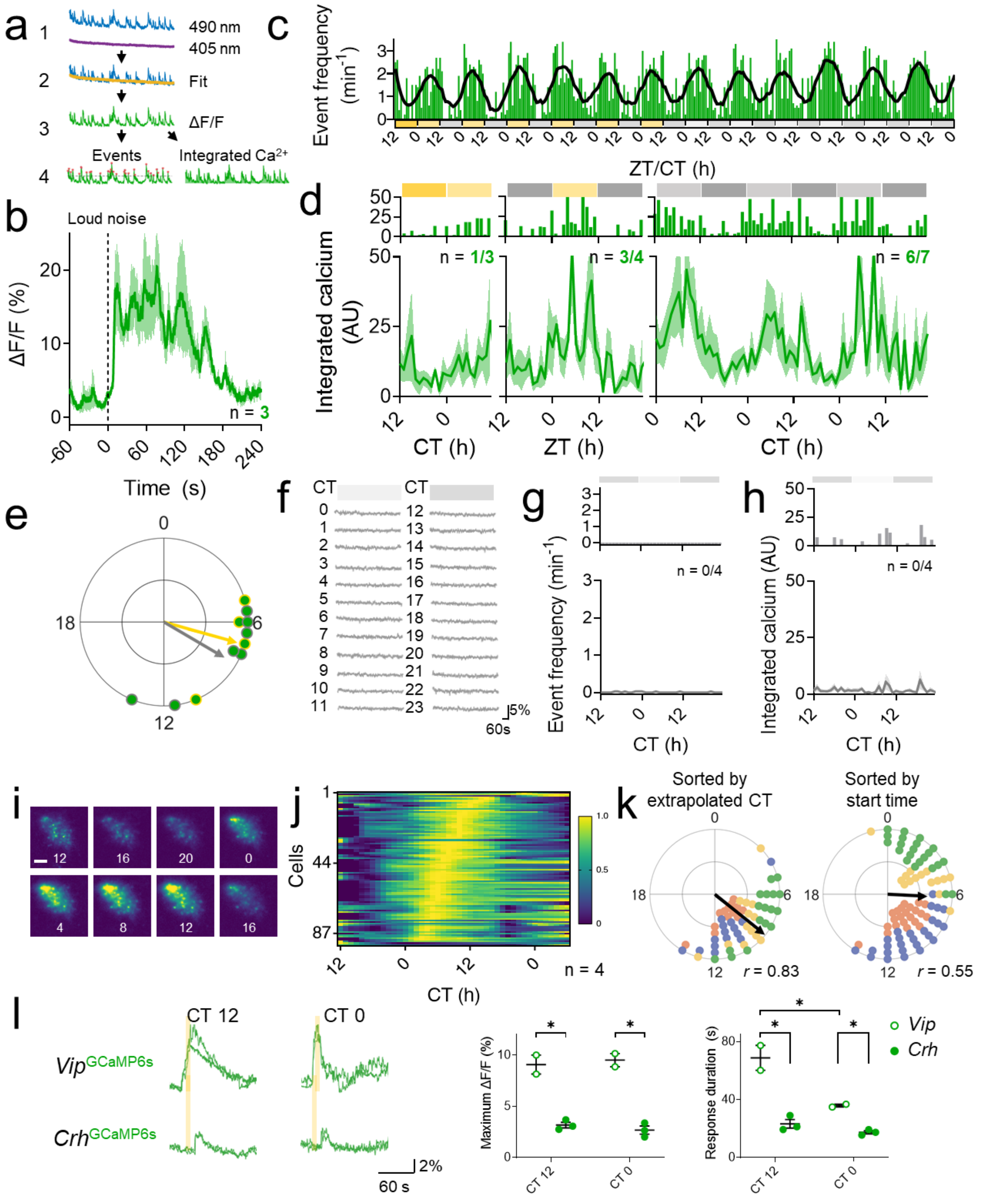

Supplementary Figure 2, related to Figure 2. Circadian PVN^{CRH} neurons rapidly respond to stress, but not light. a) Schematic for fiber photometry analysis of GCaMP6s fluorescence. For details, see methods. **b**) *In vivo* GCaMP6s fluorescence in PVN^{CRH} neurons before and after presenting mice (n = 3, 2 replicates per animal) with a stressor (a loud noise). Green lines and shading depict mean ± SEM. **c**) *In vivo* PVN^{CRH} neuron calcium event frequency (green bars) from a representative mouse housed in LL, LD, and DD over 12 days.

Data were smoothed with a 4 h Savitzky-Golay filter (black line). **d**) Integrated calcium level rhythms from PVN^{CRH} neurons in mice recorded in LL (n = 1/3 mice circadian, JTK cycle, $p = 0.035$ for 1 mouse, see Supplementary Table 1 for *p* values of individual mice), LD (n = 3/4 mice rhythmic, JTK cycle, $p < 0.001$ for 3 mice, see Supplementary Table 1 for *p* values of individual mice) and in DD (n = 6/7 mice rhythmic, JTK cycle, $p < 0.042$ for 6 mice, see Supplementary Table 1 for *p* values of individual mice). *Top*, integrated calcium level rhythms from a representative mouse; *bottom*, integrated calcium level rhythms averaged from multiple mice. Green lines and shading depict mean ± SEM. **e**) Rayleigh plots of integrated calcium level rhythms in PVN^{CRH} neurons from mice housed in LD (green dots with yellow outlines; peak time ZT 7.0, Rayleigh test, $p = 0.041$) and in DD (gray outlines; peak time CT 8.0, Rayleigh test, $p = 0.012$). **f**) Representative control (EGFP) traces (in ΔF/F) from PVN^{CRH} neurons recorded hourly from a mouse in DD (where light gray = subjective day). **g**) Event frequencies from PVN^{CRH} neurons in control (EGFP) mice recorded in DD (n = 0/4 mice rhythmic, JTK Cycle, $p = 1$ for all mice). *Top*, event frequencies from a representative control mouse; *bottom*, event frequencies averaged from multiple control mice. Gray lines and shading depict mean ± SEM. **h**) Integrated fluorescence levels from PVN^{CRH} neurons in control (EGFP) mice recorded in DD (n = 0/4 mice rhythmic, JTK Cycle, $p = 1$ for all mice). *Top*, integrated fluorescence levels from a representative control mouse; *bottom*, integrated fluorescence levels averaged from multiple control mice. Gray lines and shading depict mean ± SEM. **i**) Representative PVN^{CRH} GCaMP6s fluorescence recorded from an *ex vivo* PVN slice over 36 h. Warmer colors depict greater fluorescence. Numbers depict the extrapolated circadian time (in h) of the recording. Scale bar=100 µm. **j**) Raster plot of PVN^{CRH} calcium levels recorded hourly from individual neurons (n = 94 neurons, 4 slices). Color bar depicts fluorescence intensity in arbitrary units. **k**) Rayleigh plots of calcium fluorescence rhythms in PVN^{CRH} neurons from *ex vivo* PVN slices. Each dot color represents a different PVN slice. *Left*, data sorted by extrapolated CT (peak time CT 8.5, JTK Cycle, $p < 0.001$). *Right*, the same data sorted by the start time of the recording (peak time CT 6.0, JTK Cycle, $p < 0.001$). Sorting methods resulted in significantly different mean peak times (Two-way circular ANOVA, $p < 0.001$). Individual slices had similar times of peak calcium when plotted as extrapolated CT, but differed in their peak times when data were sorted by the start time of the recording. **l**) *Left*, representative calcium fluorescence traces (in ΔF/F) from (*top*) SCN^{VIP} neurons of *Vip*-Cre + GCaMP6s (*Vip*, n = 2) mice and (*bottom*) PVN^{CRH} neurons of *Crh*-Cre + GCaMP6s (*Crh*, n = 3) mice in response to a 10 s light pulse at CTs 12 or 0. Two-way repeated measures ANOVA with post-hoc Sidak's multiple comparisons test, $^{*}p < 0.001$. *Right*, maximum calcium fluorescence and duration of responses (time until half-maximum) after a 10 s light pulse at CT 12 or 0 in *Vip* (open green circles) and *Crh* (filled green circles) mice. Two-way repeated measures ANOVA with post-hoc Sidak's multiple comparisons test, $^{*}p = 0.020$, $^{**}p < 0.001$. Source data are provided as a Source Data file.

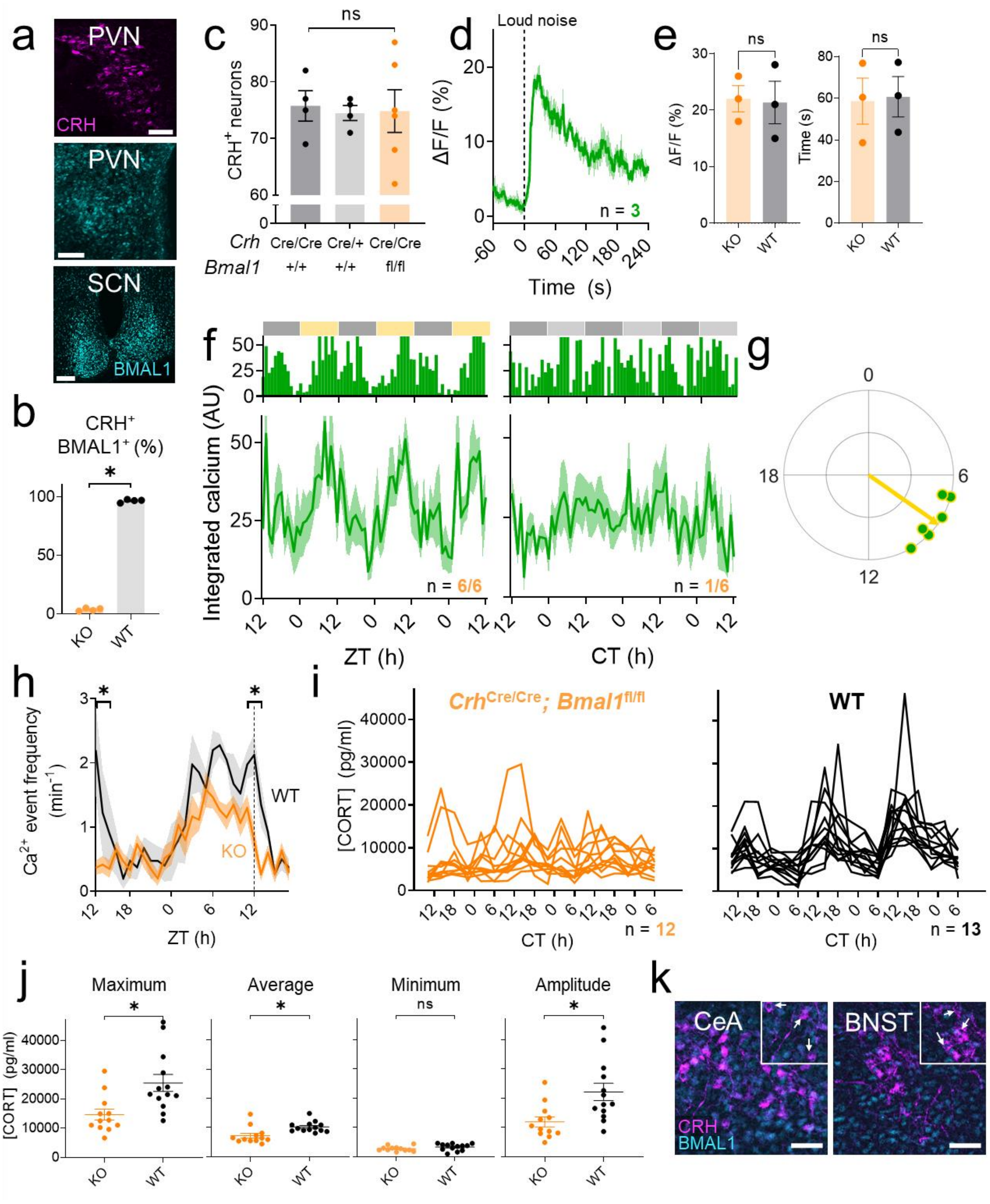

Supplementary Figure 3, related to Figure 3. Loss of BMAL1 in CRH neurons results in the loss of circadian, but not stress-induced, regulation of activity and of corticosterone release. a) Representative immunohistochemistry images from a *Crh*$^{Cre/Cre}$; *Bmal1*$^{fl/fl}$ mouse depicting CRH (magenta) and BMAL1 (cyan) expression in the PVN and SCN. Scale bar 100 µm. **b**) Percentage of CRH^{+} neurons in the PVN that co-

expressed BMAL1 in *Crh*$^{Cre/+}$; *Bmal1*$^{fl/fl}$ (WT, n = 4) and *Crh*$^{Cre/Cre}$; *Bmal1*$^{fl/fl}$ (KO, n = 4) mice. Unpaired two-tailed Welch's *t*-test, $p < 0.001$. **c**) CRH neuron numbers in the PVN of *Crh*$^{Cre/Cre}$ (n = 4), *Crh*$^{Cre/+}$ (n = 4), and *Crh*$^{Cre/Cre}$; *Bmal1*$^{fl/fl}$ mice (n = 6). Brown-Forsythe ANOVA, $p = 0.955$. In (**b-c**), mice were injected with colchicine, perfused 24 h later at CT 6, and sectioned. Brains were processed and stained identically (same antibody concentrations, durations, etc.). Images were taken using identical laser and capture settings. Lines depict mean ± SEM. **d**) *In vivo* GCaMP6s fluorescence in PVN^{CRH} neurons before and after presenting KO mice (n = 3, 2 replicates per animal) with a stressor (a loud noise). Green lines and shading depict mean ± SEM. **e**) (*Left*) Maximum calcium fluorescence and (*right*) duration of responses (time until half-maximum) in PVN^{CRH} neurons recorded from WT (n = 3) and KO (n = 3) mice after a stressor. Unpaired two-tailed Welch's *t*-test, $p = 0.889$, 0.895 for maximum fluorescence and response duration, respectively. Lines depict mean ± SEM. **f**) Integrated calcium level rhythms from PVN^{CRH} neurons in KO mice recorded in LD (n = 6/6 mice rhythmic, JTK Cycle, $p < 0.018$ for all mice, see Supplementary Table 1 for *p* values of individual mice) and in DD (n = 1/6 mice rhythmic, JTK Cycle $p < 0.001$ for 1 mouse, see Supplementary Table 1 for *p* values of individual mice). *Top*, integrated calcium level rhythms from a representative mouse; bottom, integrated calcium level rhythms averaged from multiple mice. Green lines and shading depict mean ± SEM. **g**) Rayleigh plots of integrated calcium level rhythms in PVN^{CRH} neurons from KO mice housed in LD (green dots with yellow outlines; peak time ZT 8.3, Rayleigh test, $p = 0.001$; KO vs WT peak time, Watson-Williams test $p = 0.289$). Arrhythmic recordings from KO mice housed in DD are not depicted. **h**) Calcium event frequency rhythms over the first 30 h of recording of WT (black, n = 4) and KO (orange, n = 6) mice housed in LD. Vertical dashed line at ZT 12 represents lights-off. Two-way repeated measures ANOVA with post-hoc Sidak's multiple comparisons test, *$p = 0.005$, **$p = 0.001$. Lines and shading depict mean ± SEM. **i**) Individual fecal corticosterone rhythms measured every 4 h over three days in WT (black, n = 13) and KO mice (orange, n = 12). **j**) Maximum, average, peak-trough amplitude, and minimum levels of fecal corticosterone averaged over three days in WT (black, n = 13) and KO (orange, n = 12) mice. Unpaired two-tailed Welch's *t*-test or two-tailed Mann-Whitney *U* test, $p = 0.005$, 0.002, 0.169, and 0.007 for maximum, average, minimum, and amplitude, respectively. Lines depict mean ± SEM. **k**) Representative immunohistochemistry images from *Crh*$^{Cre/Cre}$; *Bmal1*$^{fl/fl}$ mouse (n = 4) depicting CRH (magenta) and BMAL1 (cyan) expression in the central nucleus of the amygdala (CeA) and bed nucleus of the stria terminalis (BNST). Scale bar 100 µm. Inset, higher magnification image. Source data are provided as a Source Data file.

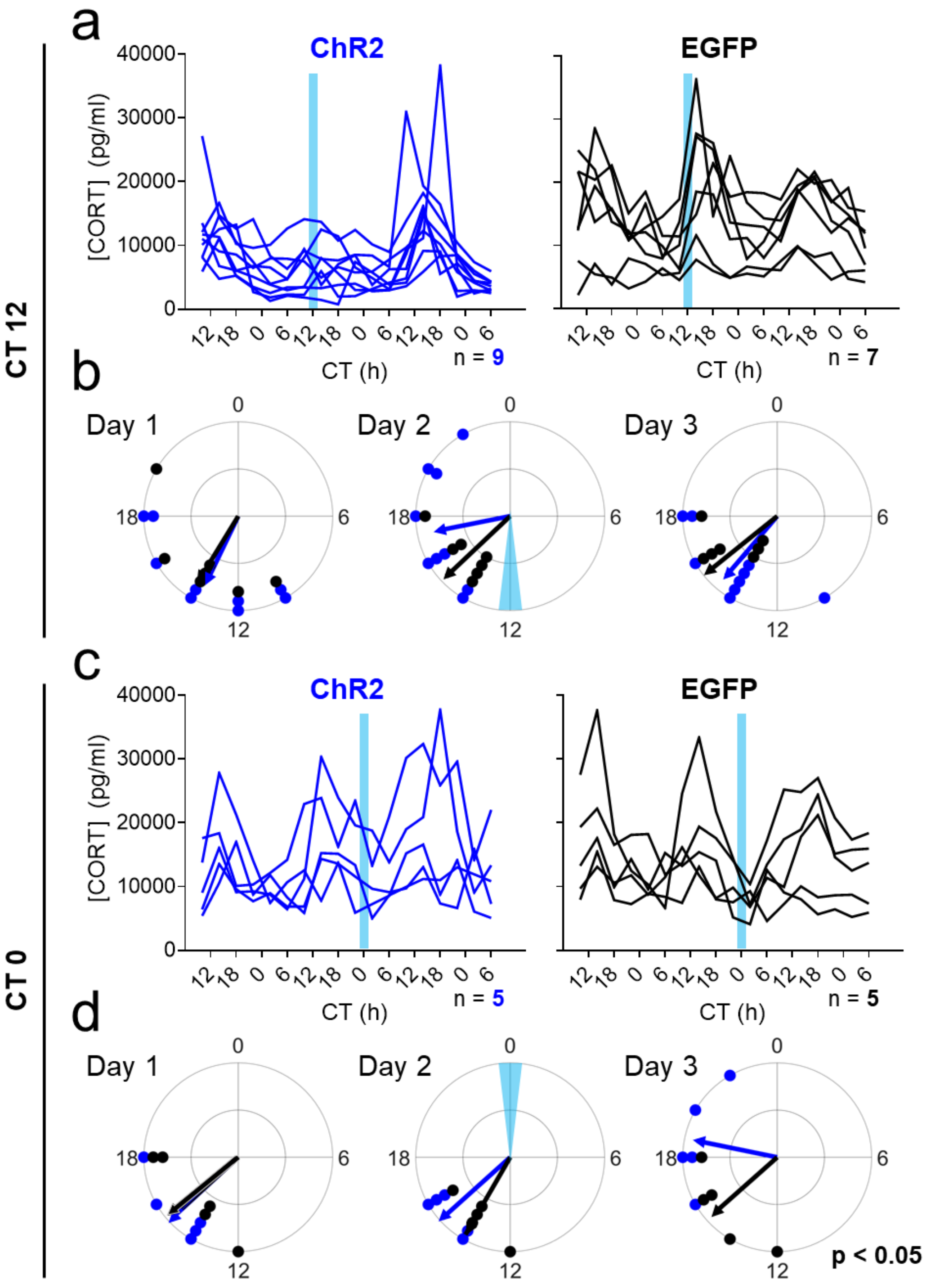

Supplementary Figure 4, related to Figure 4. Corticosterone rhythms are greatly attenuated by SCN^{VIP} neuron activation around subjective dusk. a) Individual fecal corticosterone rhythms measured every 4 h over three days in *Vip*$^{Cre/+}$ + ChR2 (ChR2, blue, n = 9) and *Vip*$^{Cre/+}$ + EGFP (EGFP, black, n = 7) mice optogenetically stimulated (blue line) on Day 2 from CT 11-13. **b**) Rayleigh plots of corticosterone rhythms in EGFP (black dots, peak times Day 1-3 CTs 14.9, 15.1 15.4, Rayleigh test, *p* = 0.014, 0.001, 0.001 on days 1, 2, and 3, respectively) and ChR2 (blue, peak times Days 1-3 CTs 13.7, 17.2, 14.7, Rayleigh test, *p* = 0.005, 0.002, 0.001 on days 1, 2, and 3, respectively) mice on each day of collection. Blue shading on Day 2 represents the time of optogenetic stimulation. **c**) Individual fecal corticosterone rhythms measured every 4 h over three days in ChR2 (blue, n = 5) and EGFP (black, n = 5) mice optogenetically stimulated (blue line) on

Day 2 from CT 23-1. **d**) Rayleigh plots of corticosterone rhythms in EGFP (black dots, peak times Days 1-3 CT 15.1, 14.1, 15.2, Rayleigh test, p = 0.028, 0.005, 0.015 on days 1, 2, and 3, respectively) and ChR2 (blue, peak times Days 1-3 CTs 15.1, 15.2, 18.7, Rayleigh test, p = 0.008, 0.003, 0.015 on days 1, 2, and 3, respectively) mice on each day of collection. Blue shading on Day 2 represents the time of optogenetic stimulation. Phases differed significantly between genotypes on Day 3 (Two-way circular ANOVA and Watson-Williams tests, p = 0.042). Source data are provided as a Source Data file.

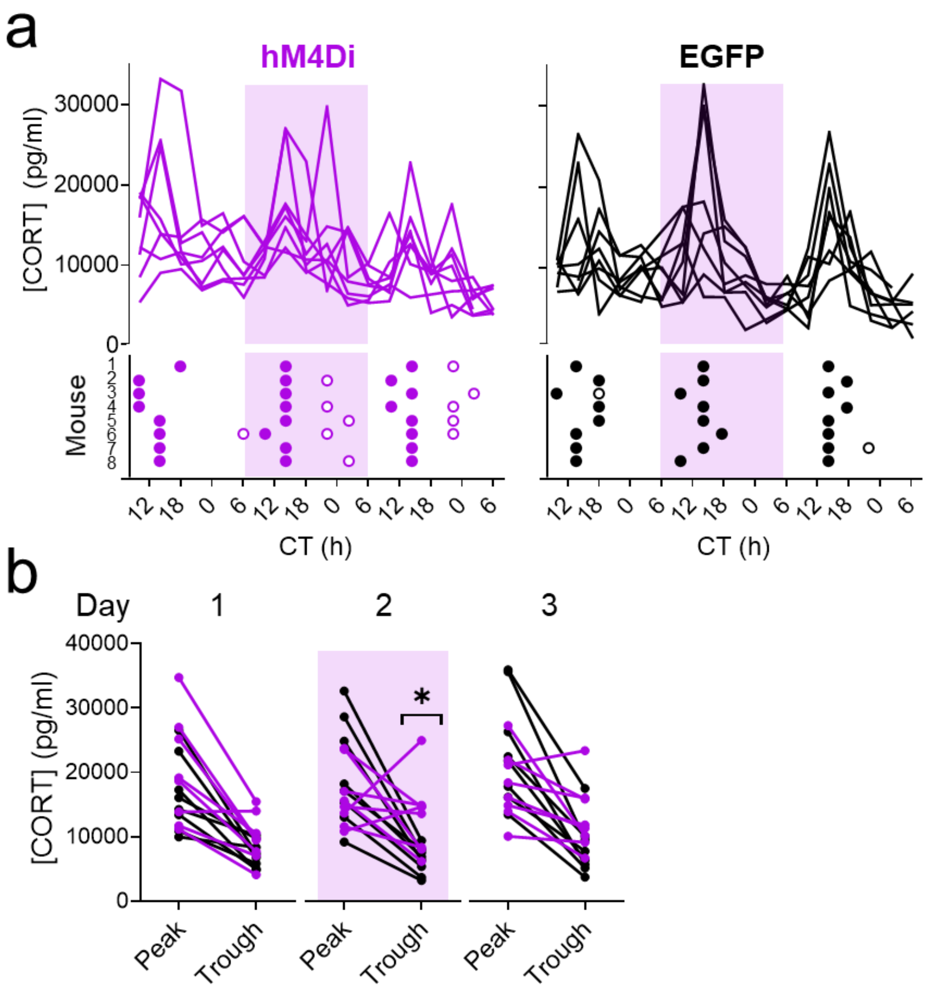

Supplementary Figure 5, related to Figure 5. Silencing SCN^{VIP} neurons produces a second peak of corticosterone release during the early morning. a) *Top,* individual fecal corticosterone rhythms measured every 4 h over three days in *Vip*$^{Cre/+}$ + hM4Di (purple, n = 8) and *Vip*$^{Cre/+}$ + EGFP (black, n = 8) mice given *ad libitum* clozapine-N-oxide (1 mg/kg) in their drinking water (purple shading) from CT 6 on Day 2 until CT 6 on Day 3. *Bottom*, peak times of corticosterone release as determined by a peak-finding algorithm in hM4Di and EGFP mice. Filled circles represent the first peak on each day, open circles represent subsequent peaks. **b**) The average peak (CT 10-18) and trough (CT 22-6) corticosterone amplitudes in EGFP (black) and hM4Di (purple) mice on each day of collection. Purple shading, time of *ad libitum* exposure to CNO in the drinking water for 24 h. Two-way repeated measures ANOVA with post-hoc Sidak's multiple comparisons test, p = 0.044. Source data are provided as a Source Data file.

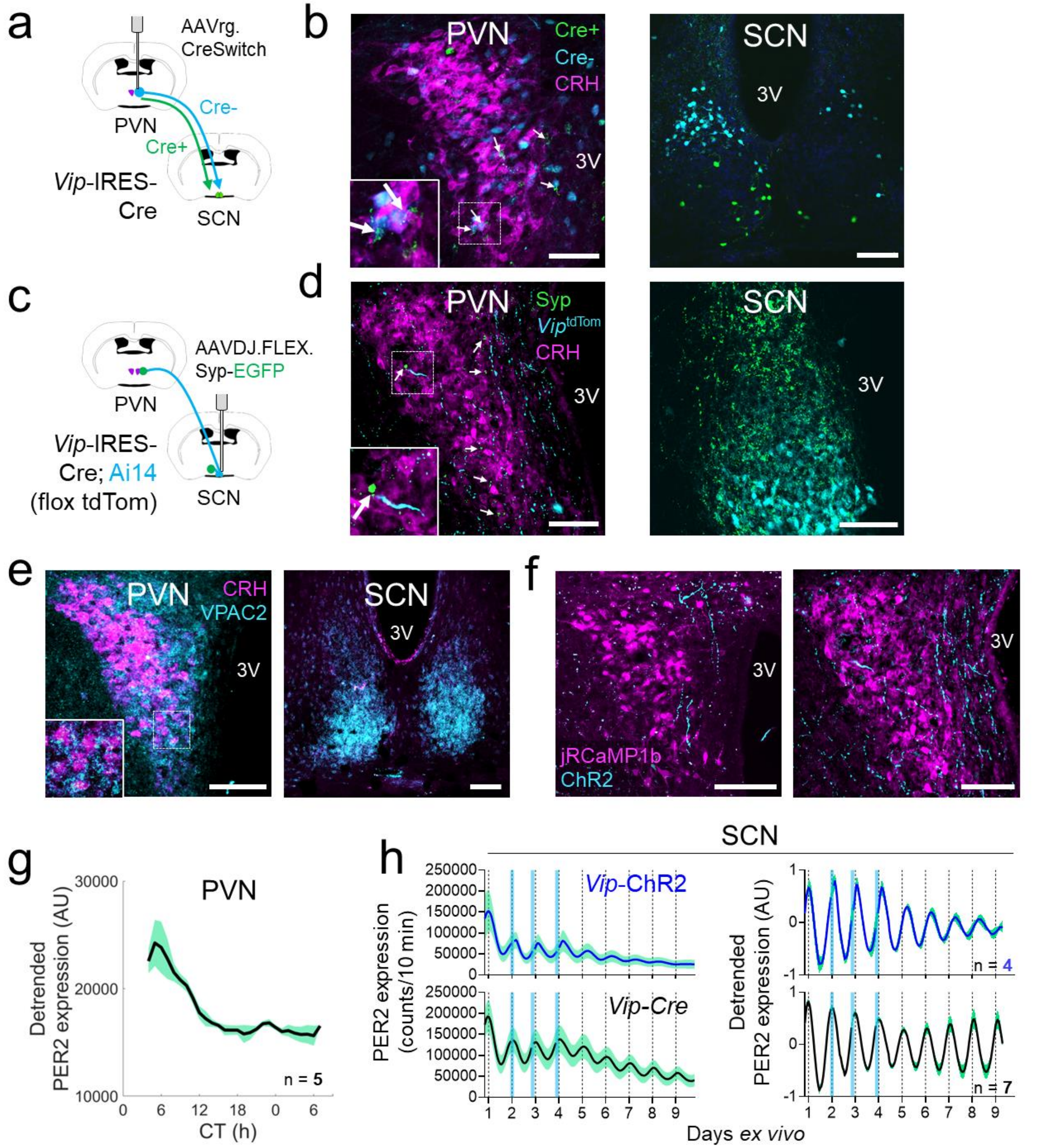

Supplementary Figure 6, related to Figure 6. Sparse monosynaptic connections and high levels of VPAC2 receptor expression suggest SCN^{VIP} neurons communicate volumetrically with PVN^{CRH} neurons. a) Schematic for retrograde monosynaptic tracing. The PVN of *Vip*$^{Cre/+}$ mice (n = 4) was unilaterally injected with a retrograde “CreSwitch” AAV that encodes tdTomato in the absence of Cre and EGFP in the presence of Cre. After transfection, starter neurons in the PVN and Cre^{-} projection neurons in the SCN would express tdTom, and *Vip*-Cre^{+} projection neurons in the SCN would express EGFP. **b**) Representative (n = 4 replicates) images of the same CreSwitch-injected mouse. (*Left*), PVN showing CRH immunoreactivity (magenta), virally-labeled Cre^{-} starter neurons (cyan), and *Vip*-Cre^{+} axons (green). Inset, higher magnification image. (*Right*), SCN showing retrograde virally-labeled Cre^{-} projection neurons (cyan) and *Vip*-Cre^{+} projection neurons (green). 3V, third ventricle. Scale bar=100 µm**. c**) Schematic for anterograde tracing. The SCN of

Vip$^{Cre/+}$; Ai14 (floxed tdTomato) mice (n = 4) was unilaterally injected with an anterograde AAV that expresses EGFP-fused synaptophysin in Cre^{+} neurons. After transfection, *Vip*-Cre^{+} axons expressing tdTomato and synaptic terminals expressing EGFP would label monosynaptic connections between the SCN and PVN. **d**) Representative (n = 4 replicates) images of the same synaptophysin-EGFP-injected mouse. (*Left*), PVN showing CRH immunoreactivity (magenta), tdTomato-expressing *Vip*-Cre^{+} axons (cyan), and virally-labeled EGFP-fused synaptophysin in *Vip*-Cre^{+} axons (green). Inset, higher magnification image. (*Right*), SCN showing tdTomato-expressing *Vip*-Cre^{+} neurons (cyan) and virally-labeled EGFP-fused synaptophysin in *Vip*-Cre^{+} axons (green). 3V, third ventricle. Scale bar=100 µm**. e**) Representative (n = 4 replicates) immunochemistry images from the same wild-type mouse depicting CRH (magenta) and the VIP receptor VPAC2 (cyan) in (*left*) the PVN and (*right*) the SCN. Inset, higher magnification image. 3V, third ventricle. Scale bar=100 µm**. f**) Representative (n = 4 replicates total) images from two additional *Vip*$^{Flp/+}$; *Crh*$^{Cre/+}$ mice showing concurrent Flp-dependent ChR2 (cyan) and Cre-dependent jRCaMP1b (magenta) expression in the PVN. 3V, third ventricle. Scale bar=100 µm**. g**) Detrended PER2::LUC bioluminescence from isolated *ex vivo* PVN slices (n = 5) over 25 h. Line and shading depict mean ± SEM. **h**) (*Left*) Raw and (*right*) detrended PER2::LUC bioluminescence traces from the SCN of *Vip*$^{Cre/+}$; PER2$^{LUC/+}$ (black, n = 6) and *Vip*$^{Cre/+}$; Ai32$^{fl/+}$; PER2$^{LUC/+}$ (blue, n = 4) mice stimulated for 1 h per day for 3 d (blue bars, 8 Hz, 470 nm, 10 ms). Source data are provided as a Source Data file.

Figure	Subject	n	Test	Result	Post-hoc test (if applicable)	Result
**1e**	LL P2V	3 mice	JTK Cycle	3/3 rhythmic; p = 0.011, 0.001, < 0.001; 95% CI [CT 4.7, 13.4]		
	LD P2V	7 mice		7/7 rhythmic; p < 0.001 for all mice; 95% CI [ZT 4.6, 7.2]		
	DD P2V	7 mice		7/7 rhythmic; p < 0.001 for all mice; 95% CI [CT 4.1, 6.5]		
**1f**	P2V amplitude	3, 7, 7 mice	Brown-Forsythe ANOVA	F(2.00, 10.74) = 8.2, p = 0.007	Dunnett's Multiple Comparisons Test	p = 0.021
**1g**	LD P2V	7 mice	Rayleigh Test	p = 0.001, z = 5.66, μ= ZT 5.9, r = 0.90		
	DD P2V	7 mice		p = 0.001, z = 5.82, μ= CT 5.3, r = 0.91		
**2d**	LL Ca2+ events	3 mice	JTK Cycle	0/3 rhythmic; p = 0.146, 0.191, 0.325		
	LD Ca2+ events	4 mice		4/4 rhythmic; p < 0.001 for all mice; 95% CI [ZT 5.7, 8.5]		
	DD Ca2+ events	7 mice		7/7 rhythmic; p = 0.030, 0.004, and < 0.001 for 5 mice; 95% CI [CT 6.3, 9.7]		
**2e**	LD Ca2+ events	4 mice	Rayleigh Test	p = 0.019, z = 3.47, μ = ZT 7.1, r = 0.93		
	DD Ca2+ events	7 mice		p = 0.003, z = 4.94, μ = CT 7.7, r = 0.84		
**3d**	KO LD Ca2+ events	6 mice	JTK Cycle	6/6 rhythmic; p = 0.034, 0.004, and < 0.001 for 4 mice; 95% CI [ZT 6.3, 8.3]		
	KO DD Ca2+ events	6 mice		0/6 rhythmic; p = 0.082, 0.070, and 1 for 4 mice		
**3e**	KO LD Ca2+ events	6 mice	Rayleigh Test	p = 0.001, z = 5.39, μ = ZT 7.3, r = 0.95		
	KO vs WT LD Ca2+ events	6, 4 mice	Watson-Williams Test	F(1, 8) = 0.07, p = 0.796		
**3f**	KO vs WT corticosterone rhythm	12, 13 mice	Mixed-effects model (REML)	Time F(17, 365) = 9.17, p < 0.001	Sidak's Multiple Comparisons Test	p = 0.041, 0.009, < 0.001
				Genotype F(1, 23) = 11.03, p = 0.003		
				Time x Genotype F(17, 365) = 3.79, p < 0.001		
**3g**	KO vs WT peak-trough amplitude	12, 13 mice	Two-Way Repeated Measures ANOVA	Genotype F(1, 23) = 32.9, p < 0.001	Sidak's Multiple Comparisons Test	p = 0.002, < 0.001
**3h**	KO corticosterone phase	12 mice	Rayleigh Test			
	Day 1			p = 0.427, z = 0.87, μ = CT 20.2, r = 0.27		
	Day 2			p = 0.101, z = 2.28, μ = CT 17.9, r = 0.43		
	Day 3			p = 0.720, z = 0.34, μ = CT 14.5, r = 0.17		
	WT corticosterone phase	13 mice	Rayleigh Test			
	Day 1			p < 0.001, z = 8.36, μ = CT 15.3, r = 0.80		
	Day 2			p < 0.001, z = 8.76, μ = CT 14.4, r = 0.82		
	Day 3			p < 0.001, z = 11.95, μ = CT 14.2, r = 0.96		
	KO vs WT	12, 13 mice	Two-Way Circular ANOVA	Genotype X^2 = x, p < 0.001		
	Day 1		Watson-Williams Test	F(1, 23) = 4.70, p = 0.040		
	Day 2			F(1, 23) = 4.36, p = 0.048		
	Day 3			F(1, 23) = 0.42, p = 0.838		
**4c**	ChR2 vs EGFP corticosterone rhythm CT 12	9, 7 mice	Mixed-effects model (REML)	Time F(17, 208) = 6.89, p < 0.001	Sidak's Multiple Comparisons Test	p < 0.001
				Genotype F(1, 14) = 7.05, p = 0.017		
				Time x Genotype F(17, 208) = 2.37, p = 0.008		
**4d**	ChR2 vs EGFP peak-trough amplitude CT 12	9, 7 mice	Two-Way Repeated Measures ANOVA	Time F(2, 28) = 7.8, p = 0.002	Sidak's Multiple Comparisons Test	p = 0.043
				Time x Genotype F(2, 28) = 7.2, p = 0.003		
**4e**	ChR2 vs EGFP corticosterone rhythm CT 0	5, 5 mice	Mixed-effects model (REML)	Time F(17, 129) = 2.21, p = 0.002		
**4f**	ChR2 vs EGFP peak-trough amplitude CT 0	5, 5 mice	Two-Way Repeated Measures ANOVA	Time x Genotype F(2, 16) = 3.53, p = 0.054		
**5c**	hM4Di vs EGFP corticosterone rhythm	8, 8 mice	Mixed-effects model (REML)	Time F(17, 224) = 10.10, p < 0.001		
				Genotype F(1, 14) = 3.95, p = 0.066		
**5d**	EGFP peak 1 phase	8 mice	Rayleigh Test			
	Day 1			p = 0.004, z = 4.88, μ = CT 15.0, r = 0.78		
	Day 2			p = 0.002, z = 5.38, μ = CT 13.5, r = 0.82		
	Day 3			p < 0.001, z = 6.50, μ = CT 14.9, r = 0.90		
	hM4Di peak 1 phase	8 mice	Rayleigh Test			
	Day 1			p = 0.004, z = 4.88, μ = CT 12.9, r = 0.78		
	Day 2			p < 0.001, z = 7.13, μ = CT 13.5, r = 0.94		
	Day 3			p < 0.001, z = 6.50, μ = CT 13.1, r = 0.90		
	hM4Di peak 2 phase	8 mice	Rayleigh Test			
	Day 2			p = 0.013, z = 3.80, μ = CT 23.6, r = 0.87		
	Day 3			p = 0.007, z = 4.20, μ = CT 22.7, r = 0.92		
	EGFP vs hM4Di	8, 8 mice	Two-Way Circular ANOVA	Genotype F(1, 42) = 3.61, p > 0.05		
				Time F(2, 42) = 0.52, p > 0.05		
	Day 1		Watson-Williams Test	F(1, 14) = 2.23, p > 0.05		
	Day 2			F(1, 14) = 0.04, p > 0.05		
	Day 3			F(1, 14) = 4.01, p > 0.05		
	Day 2 hM4Di peak 1 vs 2	8, 8 mice	Watson-Williams Test	F(1, 11) = 80.68, p < 0.001		
	Day 3 hM4Di peak 1 vs 2			F(1, 11) =72.27, p < 0.001		
**5e**	hM4Di vs EGFP peak-trough amplitude	8, 8 mice	Two-Way Repeated Measures ANOVA	Genotype F(1, 14) = 15.98, p = 0.001	Sidak's Multiple Comparisons Test	p = 0.010, 0.006
**6e**	Pre vs post stim Ca2+ events	4 slices (102 cells)	Nested One-Way ANOVA between slices	F(1, 6) = 2.90, p = 0.140		
			Two-Way Repeated Measures ANOVA	Time F(1, 101) = 66.52, p < 0.001		
	Pre vs post stim integrated Ca2+	4 slices (102 cells)	Nested One-Way ANOVA between slices	F(1, 6) = 0.81, p = 0.403		
			Two-Way Repeated Measures ANOVA	Time F(1, 101) = 57.21, p < 0.001		
**6h**	Before vs during CNO Ca2+ events	3 slices (151 cells)	Nested One-Way ANOVA between slices	F(1, 4) = 0.21, p = 0.673		
			Two-Way Repeated Measures ANOVA	Time F(1, 160) = 0.89, p = 0.345		
	Before vs during CNO integrated Ca2+	3 slices (151 cells)	Nested One-Way ANOVA between slices	F(1, 4) = 0.01, p = 0.976		
			Two-Way Repeated Measures ANOVA	Time F(1, 160) = 55.20, p < 0.001		
**6j**	SCN vs PVN, ChR2 vs EGFP P2L rhythms	6, 4 slices	Two-Way Circular ANOVA			
	Day 1			Region F(1, 18) = 0.40, p > 0.05		
				Treatment F(1, 18) = 0.05, p > 0.05		
	Day 2			Region F(1, 18) = 0.86, p > 0.05		
				Treatment F(1, 18) = 4.11 p > 0.05		
	Day 3			Region F(1, 18) = 0.18, p > 0.05		
				Treatment F(1, 18) = 10.12, p > 0.05		
	Day 4			Region F(1, 18) = 0.11, p > 0.05		
				Treatment F(1, 18) = 33.71, p < 0.001		
	Day 5			Region F(1, 18) = 4.26, p > 0.05		
				Treatment F(1, 18) = 79.28, p < 0.001		
	Day 6			Region F(1, 18) = 0.90, p > 0.05		
				Treatment F(1, 18) = 59.58, p < 0.001		
	Day 7			Region F(1, 18) = 1.17, p > 0.05		
				Treatment F(1, 18) = 52.23, p < 0.001		
	Day 8			Region F(1, 18) = 2.89, p > 0.05		
				Treatment F(1, 18) = 79.12, p < 0.001		

**S2d**	LL integrated Ca2+	3 mice	JTK Cycle	1/3 rhythmic; p = 0.035, 1 for 2 mice		
	LD integrated Ca2+	4 mice		3/4 rhythmic; p = 0.212, < 0.001 for 3 mice; 95% CI [ZT 5.1, 9.1]		
	DD integrated Ca2+	7 mice		6/7 rhythmic; p = 0.185, 0.042, 0.005, 0.001, and < 0.001 for 3 mice; 95% CI [CT 6.2, 10.4]		
**S2e**	LD integrated Ca2+	4 mice	Rayleigh Test	p = 0.041, z =2.96, µ = ZT 7.0, r = 0.86		
	DD integrated Ca2+	7 mice		p = 0.012, z = 4.02, µ = CT 8.0, r = 0.76		
**S2g**	EGFP Ca2+ events	4 mice	JTK Cycle	0/4 rhythmic, p = 1 for all mice		
**S2h**	EGFP integrated Ca2+	4 mice	JTK Cycle	0/4 rhythmic, p = 1 for all mice		
**S2k**	In vitro Ca2+ (CT sort)	94 cells, 4 slices	Rayleigh test	p < 0.001, z = 51.82, µ = CT 8.5, r = 0.83		
	In vitro Ca2+ (start time sort)			p < 0.001, z = 25.59, µ = CT 6.0, r = 0.55		
	CT vs start time sort		Two-Way Circular ANOVA	Sort X^2 = 40.56, p < 0.001		
				Sort x Slice X^2 = 39.29, p < 0.001		
			Watson-Williams Test	CT sort F(2, 21) = 1.28, p > 0.05		
				Start time sort F(2, 21) = 5.63, p = 0.010		
**S2l**	VIP vs CRH maximum dFF	2, 3 mice	Two-Way Repeated Measures ANOVA	Genotype F(1, 3) = 543, p < 0.001	Sidak's Multiple Comparisons Test	p < 0.001
	VIP vs CRH response duration			Genotype F(1, 3) = 62.86, p < 0.01	Sidak's Multiple Comparisons Test	p = 0.020, < 0.001
				Time F(1, 3) = 29.41, p = 0.01		
				Time x Genotype F(1, 3) = 14.38, p < 0.05		
**S3b**	KO vs WT CRH+ BMAL+ neurons	4, 4 slices	Unpaired Welch's *t*-test (Two-Tailed)	t(5.99) = 102.2, p < 0.001		
**S3c**	Cre/Cre vs Cre/+ vs Cre/Cre fl/fl CRH neurons	4, 4, 6 slices	Brown-Forsythe ANOVA	F(2.00, 8.84) = 0.05, p = 0.955		
**S3e**	KO vs WT maximum dFF	3, 3 mice	Unpaired Welch's t-test (Two-Tailed)	t(3.32) = 0.15, p = 0.889		
	KO vs WT response duration			t(3.93) = 0.14, p = 0.895		
**S3f**	KO LD integrated Ca2+	6 mice	JTK Cycle	6/6 rhythmic; p = 0.018, 0.002, 0.005, and < 0.001 for 3 mice; 95% CI [ZT 7.4, 9.2]		
	KO DD integrated Ca2+	6 mice		1/6 rhythmic; p = 0.192, 0.234, 0.687, < 0.001, and 1 for 2 mice		
**S3g**	KO LD integrated Ca2+	6 mice	Rayleigh Test	p = 0.001, z = 5.51, µ = ZT 8.3, r = 0.96		
	KO DD integrated Ca2+	6 mice		p > 0.05, z = 0.76, µ = CT 9.1, r = 0.36		
	KO vs WT LD intg. Ca2+	6, 4 mice	Watson-Williams Test	F(1, 8) = 1.29, p = 0.289		
**S3h**	KO vs WT Ca2+ events	6, 4 mice	Two-Way Repeated Measures ANOVA	Time F(48, 358) = 8.30, p < 0.001<br>Genotype F(1, 8) = 9.56, p = 0.020<br>Time x Genotype F(48, 358) = 2.32, p < 0.001	Sidak's Multiple Comparisons Test	p = 0.005, 0.001
**S3j**	KO vs WT corticosterone	12, 13 mice				
	Maximum		Unpaired Welch's t-test (Two-Tailed)	t(20.26) = 3.14, p = 0.005		
	Minimum			t(21.75) = 1.42, p = 0.169		
	Amplitude			t(18.97) = 3.02, p = 0.007		
	Average		Mann-Whitney *U* test (Two-Tailed)	*U* = 23, p = 0.002		
**S4b**	EGFP corticosterone phase	7 mice	Rayleigh Test			
	Day 1			p = 0.014, z = 4.87, µ = CT 14.9, r = 0.74		
	Day 2			p = 0.001, z = 5.39, µ = CT 15.1, r = 0.77		
	Day 3			p = 0.001, z = 6.24, µ = CT 15.4, r = 0.83		
	ChR2 corticosterone phase	9 mice	Rayleigh Test			
	Day 1			p = 0.005, z = 3.91, µ = CT 13.7, r = 0.75		
	Day 2			p = 0.002, z = 6.05, µ = CT 17.2, r = 0.93		
	Day 3			p = 0.001, z = 6.11, µ = CT 14.7, r = 0.93		
	EGFP vs ChR2	9, 7 mice	Two-Way Circular ANOVA	Day F(1, 42) = 2.94, p = 0.061		
	Day 1		Watson-Williams Test	F(1, 14) = 0.06, p > 0.05		
	Day 2			F(1, 14) = 3.20, p = 0.090		
	Day 3			F(1, 14) = 0.47, p > 0.05		
**S4d**	EGFP corticosterone phase	5 mice	Rayleigh Test			
	Day 1			p = 0.028, z = 3.30, µ = CT 15.1, r = 0.81		
	Day 2			p = 0.005, z = 4.48, µ = CT 14.1, r = 0.95		
	Day 3			p = 0.015, z = 3.73, µ = CT 15.2, r = 0.86		
	ChR2 corticosterone phase	5 mice	Rayleigh Test			
	Day 1			p = 0.008, z = 4.19, µ = CT 15.1, r = 0.92		
	Day 2			p = 0.003, z = 4.68, µ = CT 15.2, r = 0.97		
	Day 3			p = 0.015, z = 3.73, µ = CT 18.7, r = 0.86		
	EGFP vs ChR2	5, 5 mice	Two-Way Circular ANOVA	Genotype F(1, 24) = 3.57, p = 0.042		
	Day 1		Watson-Williams Test	F(1, 8) = 0.001, p > 0.05		
	Day 2			F(1, 8) = 2.27, p > 0.05		
	Day 3			F(1, 8) = 5.87, p = 0.041		
**S5b**	EGFP vs hM4Di CORT amplitude	8, 8 mice	Two-Way Repeated Measures ANOVA	Time x Genotype F(5, 70) = 2.524, p = 0.037	Sidak's Multiple Comparisons Test	p = 0.044

Supplementary Table 1. Statistical tests and results for each relevant figure in the paper.

Genotype	Primer	Sequence
***Crh*-IRES-Cre**	Mutant forward	CAA TGT ATC TTA TCA TGT CTG GAT CC
	Common	CTT ACA CAT TTC GTC CTA GCC
	Wild-type forward	CAC GAC CAG GCT GCG GCT AAC
Bmal1fl/fl	Forward	ACT GGA AGT AAC TTT ATC AAA CTG
	Reverse	CTG ACC AAC TTG CTA ACA ATT A
***Vip*-IRES-Cre**	Mutant forward	CCC CCT GAA CCT GAA ACA TA
	Common	GGA CAC AGT AAG GGC ACA CA
	Wild-type forward	TCC TTG GAA CAT TCC TCA GC
**Ai14**	Wild-type forward	AAG GGA GCT GCA GTG GAG TA
	Wild-type reverse	CCG AAA ATC TGT GGG AAG TC
	Mutant forward	GGC ATT AAA GCA GCG TAT CC
	Mutant reverse	CTG TTC CTG TAC GGC ATG G
***Vip*-IRES-Flp**	Mutant forward	AGG ATT GGG AAG ACA ATA GCA
	Common	CAC CTC TGA TTT CAG CTC TGC
	Wild-type forward	GGC TGA TTT TCA ATA GTA TGG TCT C
**Ai32**	Wild-type forward	AAG GGA GCT GCA GTG GAG TA
	Wild-type reverse	CCG AAA ATC TGT GGG AAG TC
	Mutant forward	ACA TGG TCC TGC TGG AGT TC
	Mutant reverse	GGC ATT AAA GCA GCG TAT CC
**PER2::LUC**	Common	CTG TGT TTA CTG CGA GAG T
	Wild-type reverse	GGG TCC ATG TGA TTA GAA AC
	Mutant reverse	TAA AAC CGG GAG GTA GAT GAG A

Supplementary Table 2. Genotyping primers used in this study.
