# Supplementary material for: Circadian neurons in the paraventricular nucleus entrain and sustain daily rhythms in glucocorticoids
Source: Nat Commun. 2021 Oct 1;12:5763. doi: 10.1038/s41467-021-25959-9 (PMC8486846; doi:10.1038/s41467-021-25959-9)
Supplement: Supplementary file 3 — Reporting Summary [file 41467_2021_25959_MOESM3_ESM.pdf]

nature research

Corresponding author(s): Erik Herzog

Last updated by author(s): 2021-08-11

Reporting Summary

Nature Research wishes to improve the reproducibility of the work that we publish. This form provides structure for consistency and transparency in reporting. For further information on Nature Research policies, see our Editorial Policies and the Editorial Policy Checklist.

Statistics

For all statistical analyses, confirm that the following items are present in the figure legend, table legend, main text, or Methods section.

n/a	Confirmed	
☐	☒	The exact sample size (*n*) for each experimental group/condition, given as a discrete number and unit of measurement
☐	☒	A statement on whether measurements were taken from distinct samples or whether the same sample was measured repeatedly
☐	☒	The statistical test(s) used AND whether they are one- or two-sided<br>*Only common tests should be described solely by name; describe more complex techniques in the Methods section.*
☒	☐	A description of all covariates tested
☐	☒	A description of any assumptions or corrections, such as tests of normality and adjustment for multiple comparisons
☐	☒	A full description of the statistical parameters including central tendency (e.g. means) or other basic estimates (e.g. regression coefficient) AND variation (e.g. standard deviation) or associated estimates of uncertainty (e.g. confidence intervals)
☐	☒	For null hypothesis testing, the test statistic (e.g. F, t, r) with confidence intervals, effect sizes, degrees of freedom and P value noted<br>*Give P values as exact values whenever suitable.*
☒	☐	For Bayesian analysis, information on the choice of priors and Markov chain Monte Carlo settings
☒	☐	For hierarchical and complex designs, identification of the appropriate level for tests and full reporting of outcomes
☒	☐	Estimates of effect sizes (e.g. Cohen's *d*, Pearson's *r*), indicating how they were calculated

Our web collection on statistics for biologists contains articles on many of the points above.

Software and code

Policy information about availability of computer code

Data collection	Behavioral data was collected with Clocklab v6.1 (Actimetrics). Fiber photometry imaging data was collected using previously-published custom Matlab (R2019 and R2020, Mathworks) code (Jones et al. 2018, available from the corresponding author upon reasonable request). Ex vivo fluorescence data was collected using a Micro-Manager script. In vitro fluorescence data was collected with QCapture and CamStudio software (QImaging). Bioluminescence data was collected using Andor software.
Data analysis	Fiber photometry imaging data was analyzed using previously-published custom Matlab code (Jones et al. 2018, available from the author upon reasonable request). Ex vivo fluorescence data was analyzed using ImageJ and a previously-published custom Python script (Tso et al. 2017, available from the author upon reasonable request). Bioluminescence data was analyzed using ImageJ and Matlab. Statistics were performed using Prism 8.0 (Graphpad) or the Circular Statistics Toolbox in Matlab. JTK analysis was performed in R (v4.2) using publicly available, previously published code (Hughes et al. 2010). Peak finding was performed in Matlab using the built-in "findpeaks" function.

For manuscripts utilizing custom algorithms or software that are central to the research but not yet described in published literature, software must be made available to editors and reviewers. We strongly encourage code deposition in a community repository (e.g. GitHub). See the Nature Research guidelines for submitting code & software for further information.

Data

Policy information about availability of data

All manuscripts must include a data availability statement. This statement should provide the following information, where applicable:

- Accession codes, unique identifiers, or web links for publicly available datasets
- A list of figures that have associated raw data
- A description of any restrictions on data availability

All data generated in this study that support our findings are presented within this paper, its supplementary materials, or in the source data. All additional information will be made available upon reasonable request to the corresponding author. Source data are provided with this paper.

Field-specific reporting

Please select the one below that is the best fit for your research. If you are not sure, read the appropriate sections before making your selection.

☒ Life sciences ☐ Behavioural & social sciences ☐ Ecological, evolutionary & environmental sciences

For a reference copy of the document with all sections, see nature.com/documents/nr-reporting-summary-flat.pdf

Life sciences study design

All studies must disclose on these points even when the disclosure is negative.

Sample size	We chose sample sizes to be sufficient for statistical analysis based on similar techniques used in previous publications (Jones et al. 2018, Mazuski et al. 2018).
Data exclusions	For in vivo data, we excluded animals from analysis if the 490 nm and 405 nm signals simultaneously fluctuated by ~100 mV (typically indicating fiber placement inside the third ventricle), if they did not exhibit circadian locomotor activity before imaging, or if the fiber or virus was off-target as determined by histology. For corticosterone data, we excluded animals from analysis if their feces collectors became clogged at any time during the course of the experiment. For in vitro data, we excluded from analysis a subset of slices that had good jRCaMP1b expression (healthy cells, no nuclear filling) but exhibited no calcium dynamics before or after stimulation (dFFpre and dFFpost = 0 +/- 0.01).
Replication	All experiments were performed with multiple groups of mice depending on the number of experimental setups available for each experiment. Immunostaining was replicated in at least 4 mice. All replicates generated similar results. Specific details for each experiment are described in the text and figure legends.
Randomization	Mice were randomly assigned to experimental groups when possible (e.g., EGFP vs ChR2). For corticosterone collection experiments, animals were randomly assigned to feces collector cages and to the location of their cage in the behavior tent.
Blinding	We had no specific methods to blind investigators to genotype or condition during the experiments themselves. For corticosterone experiments, all animals received identical treatments at the same time of day. Investigators were blinded to the genotype or condition when processing data (e.g., corticosterone samples). We performed data analysis blind to genotype or condition.

Reporting for specific materials, systems and methods

We require information from authors about some types of materials, experimental systems and methods used in many studies. Here, indicate whether each material, system or method listed is relevant to your study. If you are not sure if a list item applies to your research, read the appropriate section before selecting a response.

Materials & experimental systems

n/a	Involved in the study	
☐	☒	Antibodies
☒	☐	Eukaryotic cell lines
☒	☐	Palaeontology and archaeology
☐	☒	Animals and other organisms
☒	☐	Human research participants
☒	☐	Clinical data
☒	☐	Dual use research of concern

Methods

n/a	Involved in the study	
☒	☐	ChIP-seq
☒	☐	Flow cytometry
☒	☐	MRI-based neuroimaging

Antibodies

Antibodies used

goat anti-cFOS, sc52-G, Santa Cruz Biotechnology
guinea pig anti-BMAL1, AB2204, Sigma
rabbit anti-CRH, ab8901, Abcam
guinea pig anti-CRH, T-5007, Peninsula Labs
chicken anti-GFP, GFP-1020, Aves Labs
rabbit anti-mCherry, ab167453, Abcam
rabbit anti-VPAC2, ab28624, Abcam
DyLight 488 donkey anti-goat, SA5-10086, Thermo Fisher
DyLight 594 donkey anti-goat, SA5-10088, Thermo Fisher
Alexa 594 donkey anti-guinea pig, 706-585-148, Jackson Immunoresearch
Alexa 488 donkey anti-rabbit, 711-545-152, Jackson Immunoresearch
Alexa 555 goat anti-rabbit, ab150078, Abcam
Alexa 488 donkey anti-chicken, 703-545-155, Jackson Immunoresearch
Alexa 647 goat anti-guinea pig, ab150187, Abcam

Validation	All antibodies listed above have been validated by the suppliers with relevant images on their websites. Additionally, all antibodies used have been cited multiple times as listed on the manufacturer's website.<br><br>Specifically, for the primary antibodies used in this paper,<br><br>sc52-G: Manufacturer's validation: datasheets.scbt.com/sc-52 , relevant citations in mouse: Jones et al. J Neurosci 2018, Fu et al. Cell Rep 2019, Pomrenze et al. Cell Rep 2019, > 100 others.<br><br>AB2204: Manufacturer's validation: emdmillipore.com/US/en/product/Anti-BMAL1-Antibody,MM_NF-AB2204, relevant citations in mouse: Tonsfeldt et al. J Endocr Soc 2019, Kobayashi et al. Neuron 2015, LeSauter et al. PLos One 2012<br><br>ab8901: Manufacturer's validation: abcam.com/crf-antibody-ab8901.html, relevant citations in mouse: Companion and Thiele Eur J Neurosci 2018, Hu et al. J Neurosci 2020, Zhang et al. Sci Sign 2020, >20 others<br><br>T-5007: Manufacturer's validation: bma.ch/files/product/t-5007.pdf, relevant citations in mouse: Hupalo et al. J Neurosci 2019, Wyrofsky et al. Eur J Neurosci 2018, Baracz et al. J Neuroendoc 2020, >30 others<br><br>GFP-1020: Manufacturer's validation: aveslabs.com/products/anti-green-fluorescent-protein-antibody-gfp, relevant citations in mouse: Zhang et al. Cell Rep 2019, Teixeira et al. J Neuroendoc 2019, ,Takai et al. PLoS One 2019, >1,000 others<br><br>ab167453: Manufacturer's validation: abcam.com/mcherry-antibody-ab167453.html, relevant citations in mouse: Hancock et al. Cell 2019, Zerbi et al. Neuron 2019, Gupta et al. 2018, , >400 others.<br><br>ab28624: Manufacturer's validation: abcam.com/vpac2-antibody-ab28624.html, relevant citations in mouse: Jones et al. J Neurosci 2018, An et al. J Comp Neurol 2012, Shan et al. Neuron 2020, 8 others

Animals and other organisms

Policy information about studies involving animals; ARRIVE guidelines recommended for reporting animal research

Laboratory animals	Mice, C57BL/6JN background, males, 2-5 months
Wild animals	Study did not involve wild animals
Field-collected samples	Study did not involve samples collected from the field
Ethics oversight	All experiments were performed in accordance with Washington University's Institutional Animal Care and Use Committee guidelines.

Note that full information on the approval of the study protocol must also be provided in the manuscript.
